# HCV co-infection in HIV positive population in British Columbia, Canada

**DOI:** 10.1186/1471-2458-10-225

**Published:** 2010-04-29

**Authors:** Jane A Buxton, Amanda Yu, Paul H Kim, John J Spinelli, Margot Kuo, Maria Alvarez, Mark Gilbert, Mel Krajden

**Affiliations:** 1 BC Centre for Disease Control, Vancouver, British Columbia; 2 School of Population and Public Health, University of British Columbia, Vancouver, British Columbia; 3 BC Cancer Agency, Vancouver, British Columbia

## Abstract

**Background:**

As HIV and hepatitis C (HCV) share some modes of transmission co-infection is not uncommon. This study used a population-based sample of HIV and HCV tested individuals to determine the prevalence of HIV/HCV co-infection, the sequence of virus diagnoses, and demographic and associated risk factors.

**Methods:**

Positive cases of HIV were linked to the combined laboratory database (of negative and positive HCV antibody results) and HCV reported cases in British Columbia (BC).

**Results:**

Of 4,598 HIV cases with personal identifiers, 3,219 (70%) were linked to the combined HCV database, 1,700 (53%) of these were anti-HCV positive. HCV was diagnosed first in 52% of co-infected cases (median time to HIV identification 3 1/2 years). HIV and HCV was diagnosed within a two week window in 26% of cases. Among individuals who were diagnosed with HIV infection at baseline, subsequent diagnoses of HCV infection was independently associated with: i) intravenous drug use (IDU) in males and females, Hazard Ratio (HR) = 6.64 (95% CI: 4.86-9.07) and 9.76 (95% CI: 5.76-16.54) respectively; ii) reported Aboriginal ethnicity in females HR = 2.09 (95% CI: 1.34-3.27) and iii) males not identified as men-who-have-sex-with-men (MSM), HR = 2.99 (95% CI: 2.09-4.27).

Identification of HCV first compared to HIV first was independently associated with IDU in males and females OR = 2.83 (95% CI: 1.84-4.37) and 2.25 (95% CI: 1.15-4.39) respectively, but not Aboriginal ethnicity or MSM. HIV was identified first in 22%, with median time to HCV identification of 15 months;

**Conclusion:**

The ability to link BC public health and laboratory HIV and HCV information provided a unique opportunity to explore demographic and risk factors associated with HIV/HCV co-infection. Over half of persons with HIV infection who were tested for HCV were anti-HCV positive; half of these had HCV diagnosed first with HIV identification a median 3.5 years later. This highlights the importance of public health follow-up and harm reduction measures for people identified with HCV to prevent subsequent HIV infection.

## Background

HIV and hepatitis C (HCV) are major burdens on the health care system in Canada and share some common modes of transmission. HCV co-infection is estimated to occur in 20% of Canadians infected with HIV [1] and 50-90% of HIV-positive persons who use drugs intravenously [2-4]. Compared to HIV mono-infected individuals, HIV/HCV co-infected groups are characterized by a higher prevalence of injection drug use, poverty, and psychiatric disorders [5].

Co-infection affects disease progression related to both agents [6-12] and complicates treatment [13-15]. The rate of liver cirrhosis is up to six times higher in HIV co-infected persons than HCV mono-infected [12,16-19]. Effective anti-retroviral therapies have improved the life expectancy of persons with HIV so that persons co-infected with chronic HCV survive to develop HCV cirrhosis.

Clinical management and treatment of co-infected patients is challenging and complex [13-15]. Abnormal hepatic function is one of the most common complications occurring among HIV-infected individuals receiving anti-retroviral therapy [14,20,21]. Consensus guildelines for co-infection state all HIV-infected individuals should be screened for HCV, and those with co-infection should be considered for anti-HCV treatment, even though response to anti-HCV therapy is lower in co-infected patients [14,16,20,21]. As antiretoviral hepatotoxicity may be increased in the presence of HCV infection, ideally HCV treatment should be intiated before anti-HIV therapy when HIV infection is stable [22]. However, treating HIV first is clearly indicated when CD4 lymphocyte count is very low (<200 cells/microL) [13].

**Figure 1:**
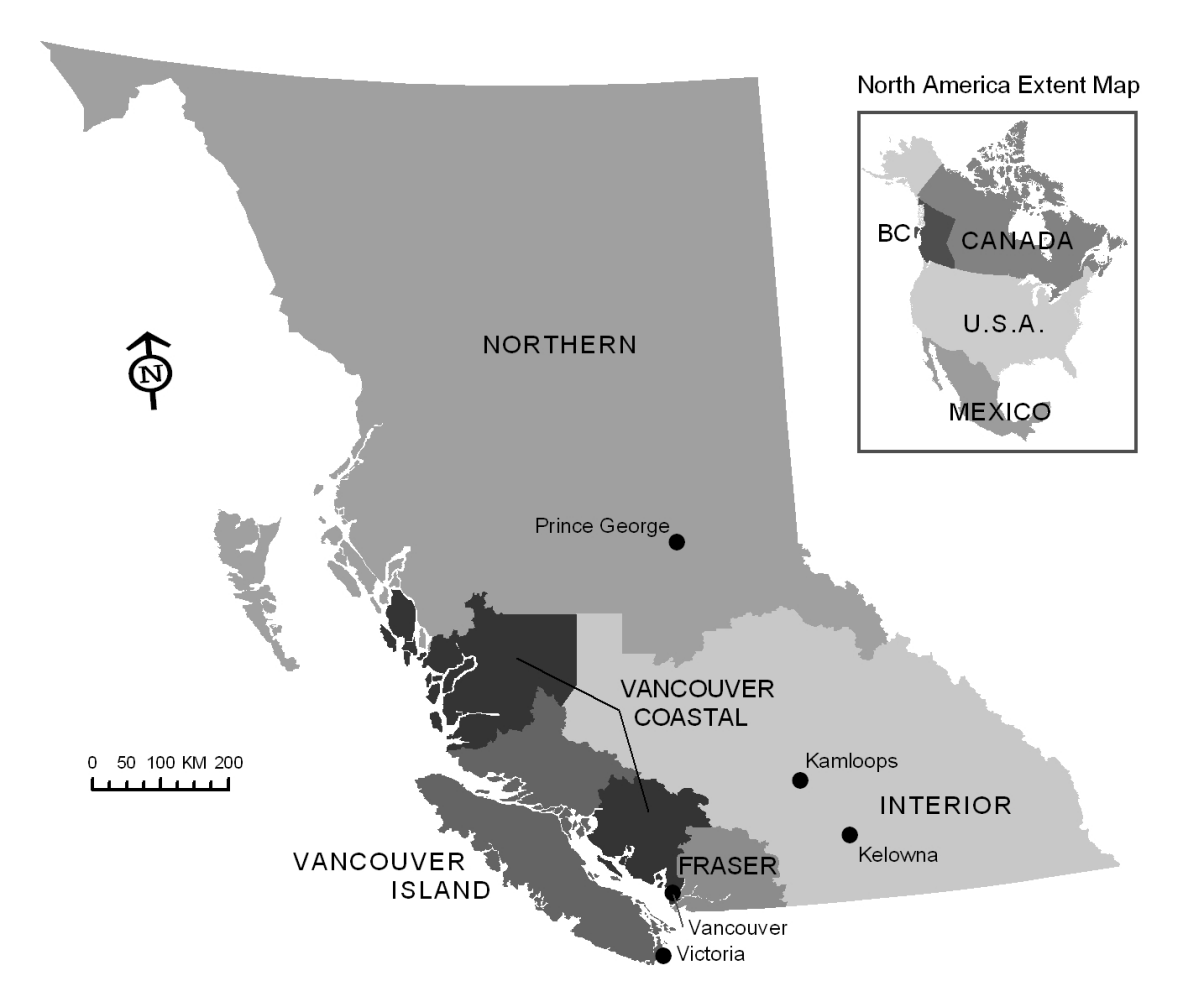
**British Columbia map of health authorities**.

Although data on HIV/HCV co-infection is available from defined study cohorts such as persons who use intravenous drugs (IDU) and men who have sex with men (MSM) there is little population-based data. All confirmatory HIV testing in British Columbia (BC) and 95% of anti-HCV testing is performed at the Provincial Public Health Reference laboratory. First positive HIV tests in BC are reported to the STI/HIV Prevention and Control Division at BC Centre for Disease Control (BCCDC). Since 1995 more than 6,000 cases of HIV have been reported, and risk factor information at the time of HIV identification collected. All newly identified cases of HCV, who are resident in BC and not previously reported elsewhere in Canada, are reported by the five regional public health authorities (Figure 1) into the BC integrated Public Health Information System (iPHIS), which is coordinated at BCCDC. Approximately 65,000 antibody reactive cases of HCV have been reported into iPHIS since 1992.

The aim of this study was to link positive cases of HIV with both anti-HCV positive cases and anti-HCV negative testers identified in the BC surveillance and laboratory systems in order to determine prevalence of HIV/HCV co-infection. The sequence of virus diagnosis, demographic characteristics and associated risk factors at the time of HIV infection were determined. This linkage enables the estimation of the current and projected burden of disease in order to guide prevention, testing and care programs, and appropriate resource allocation.

## Methods

### Data sources

The STI/HIV Prevention and Control database (HIV dataset) which contains all anti-HIV positive individuals (confirmed by HIV Western Blot) was linked to the combined BCCDC laboratory HCV testing database and reported HCV cases in iPHIS (HCV dataset). The timeframe of the data linkage was from January 1^st ^1995 to December 31^st ^2008.

HCV antibody testing was performed using second- or third-generation enzyme-linked immunosorbent assays (EIA) including Organon Teknika (UBI) v2.0, v2.1, v4.0 (Organon Teknika, Durham, North Carolina) and Abbott AxSYM HCV 3.0 (Abbott Diagnostics, Chicago, Illinois). Specimens reactive for anti-HCV were retested by the second or third generation Recombinant Immunoblot Assay (Chiron, Emeryville, California) until July 1999 to confirm EIA specificity. Between April 1997 and February 2004, Abbott AxSYM HCV 3.0 anti-HCV reactive specimens were re-tested by either Organon Teknika (UBI) v4.0 or Ortho EcI (Ortho, Canada). Thereafter, anti-HCV screening was performed on the ADVIA Centaur (initially Bayer, Canada, now Siemens, Canada) and anti-HCV reactive samples re-tested on the Abbott Architect (Abbott, Canada). Only specimens reactive by both manufacturers' tests were considered to be anti-HCV reactive. As HCV RNA test results (qualitative COBAS AMPLICOR HCV Test v2.0 (Roche Diagnostic Systems, Mississauga, Canada) were only available on a subset of HCV antibody positive cases these data were not included in our analysis. Data elements include first and last name, personal health number, date of birth, sex, location (health region of residence of the subject at the time of HIV virus identification), ethnicity (self- or clinician-reported), date each virus was identified, and exposure category(ies) at the time HIV infection was identified.

### Data management and confidentiality

The HIV and HCV datasets were linked on personal identifiers; after which nominal information was permanently deleted and a unique study code was allocated. To maintain confidentiality the linkage process was performed by computer programmed algorithms without displaying personal identifiers. All datasets used in the linkage process with retained identifiers were permanently deleted leaving only the final, linked, anonymized dataset to be used for the statistical analyses. Data manipulation and analyses were performed using SAS version 9.1.3 for Windows (SAS Institute, Cary, NC).

All first positive HIV cases, identified from January 1^st ^1995 to December 31^st ^2008 with personal identifiers, were linked with the combined HCV dataset. Those with HCV and HIV first identified within 2 weeks were considered simultaneous identification. Ethical approval was received from the UBC Clinical Research Ethics Board.

### Statistical analysis

Cases aged <15 years and non-BC residents were excluded. Univariate logistic regression was used to compare linked and unlinked subjects. Univariate and multivariate Cox proportional hazard regression were used to estimate the hazard ratios (HR) and 95% confidence intervals of HIV/HCV co-infection among individuals with HIV mono-infection at baseline. Univariate and multivariate logistic regression were used to assess the association between the sequence of virus identification and explanatory variables among co-infected individuals; odds ratios (OR) and 95% Wald confidence intervals were estimated. Multivariate regression analyses were conducted separately by sex as predictive factors were different for males and females, covariates included age, ethnicity, health authority of residence at HIV infection and IDU for both sexes; MSM was included for males.

## Results

**Figure 2:**
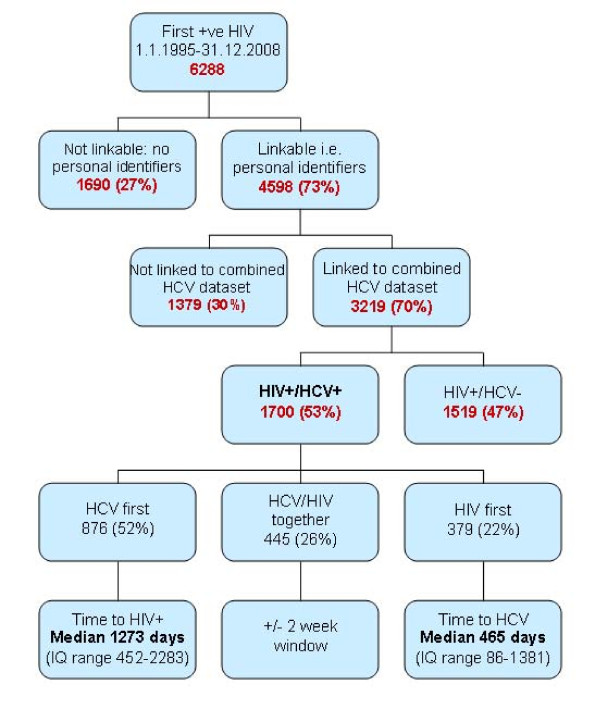
**HIV data linked with HCV dataset; January 1, 1995 to December 31, 2008**.

A total of 6,288 HIV cases were eligible for analysis; 4,598 (73%) had personal identifiers and therefore were potentially linkable to the combined HCV dataset. The HCV dataset comprised test results from 914,442 individuals, of whom 69,339 (7.6%) were anti-HCV antibody positive. In total, 3,219 (70%) HIV cases were linked to the HCV dataset (Figure 2). The HIV positive population that was linked to the HCV dataset were younger than those not linked; mean age 37.9 vs. 39.3 (t = 4.00, p < 0.0001). In univariate analysis those linked were more likely to be female (O.R. 2.01, 95% CI 1.70-2.38), Aboriginal (O.R. 1.96, 95% CI 1.60-2.41), and to not reside in Vancouver Coastal Health Region (O.R. 1.90, 95% CI 1.67-2.16).

Of the 3,219 persons with HIV that were linked to the HCV dataset, 1,700 (53%) were co-infected; i.e. at least 27% of all HIV cases. Within those co- infected, HCV was diagnosed first in 52% of cases, with a median time to subsequent HIV identification 3 1/2 years; 26% were diagnosed at about the same time (± 2 weeks); HIV was identified first in 22%, with median time to HCV identification of 15 months (Figure 2).

Among individuals who were HIV mono-infected at baseline, subsequent diagnosis of HCV (compared to persons without a HCV infection diagnosis) using univariate analysis was significantly associated with a younger age at HIV identification, reported Aboriginal ethnicity, reported IDU and not being identified as MSM in males (Table 1).

**Table 1:** HIV mono-infected cases at baseline linked to HCV dataset January 1, 1995 to December 31, 2008

	HCV not detected (%)		HCV co-infected (%)		Unadjusted HR		95% Confidence Limits	
**N**	1519		379					
								
**Age at HIV**					**0.97**		**0.96-0.98**	
Mean	38.6		34.3					
Std	11.67		9.05					
Range	15-82		15-61					
								
**Sex**								
Male	1234 (81.2)		265 (69.9)		1.00			
Female	274 (18.0)		112 (29.6)		**1.61**		**1.26-2.04**	
Transgender	1 (0.1)		1 (0.3)					
Unknown/ missing	10 (0.7)		1 (0.3)					
								
**Ethnicity**								
Non-Aboriginal	1292 (85.1)		234 (61.7)		1.00			
Aboriginal	136 (9.0)		79 (20.8)		**2.72**		**2.11-3.52**	
Unknown/ missing	91 (6.0)		66 (17.4)					
								
**Health Authority**								
Vancouver Coastal	829 (54.6)		191 (50.4)		1.00			
Fraser	432 (28.4)		108 (28.5)		1.04		0.80-1.36	
Interior	86 (5.7)		21 (5.5)		1.07		0.65-1.76	
Northern	52 (3.4)		10 (2.6)		1.16		0.59-2.27	
Vancouver Island	118 (7.8)		45 (11.9)		**1.17**		**1.23-2.40**	
Missing	2 (0.1)		4 (1.1)					

	**Male**	**Female**	**Male**	**Female**	**Male**	**Female**	**Male**	**Female**
**MSM**								
Yes	796 (64.5)	-	47 (17.7)	-	1.00			
No	438 (35.5)	-	218 (82.3)	-	**5.40**		**3.90-7.49**	-
								
**IDU**								
Yes	122 (9.9)	37 (13.5)	169 (63.8)	81 (72.3)	**10.33**	**10.94**	**7.76-13.77**	**6.61-18.10**
No	1112 (90.1)	237 (86.5)	96 (36.2)	31 (27.7)	1.00	1.00		

The Cox proportional hazard assumption was tested by including the interactions of predictors and log survival time as time dependent covariates in the model. The results suggested that the proportionality assumption of the model was not violated. In multivariable Cox regression model of individuals who were HIV mono-infected at baseline (Table 2), males aged 45+ at HIV identification were less likely to become co-infected compared to males aged 15-24, (HR 0.55, 95% CI: 0.31-0.97), but age was not significant for females. Males and females who reported IDU were more likely to become co-infected: HR 6.64, 95% CI: 4.86-9.07 and HR 9.76, 95% CI: 5.76-16.54 respectively. Females reporting Aboriginal ethnicity were twice as likely to become co-infected (HR 2.09, 95% CI: 1.34-3.27); ethnicity was not significant for males. Males not identified as MSM were more likely to become co-infected (HR 2.99, 95% CI: 2.09-4.27).

**Table 2:** Adjusted hazard ratios (Cox regression) for risk of co-infection

	Adjusted HR*		95% Confidence Limits	
	**Male** **(N = 137)**	**Female** **(N = 354)**	**Male**	**Female**

**Age group at HIV**				
25-34 years vs. 15-24 years	0.77	1.19	0.47-1.27	0.72-1.97
35-44 years vs. 15-24 years	0.78	1.55	0.48-1.27	0.89-2.70
45+ years vs. 15-24 years	**0.55**	0.99	**0.31-0.97**	0.39-2.49
**Health Authority (HA) vs. Vancouver Coastal HA**				
Fraser HA	0.81	1.06	0.58-1.12	0.62-1.81
Interior HA	0.89	1.41	0.48-1.66	0.59-3.39
Northern HA	0.73	0.51	0.32-1.69	0.16-1.68
Vancouver Island HA	1.10	**2.23**	0.70-1.71	**1.28-3.89**
**Aboriginal **Yes vs. No	1.29	**2.09**	0.88-1.88	**1.34-3.27**
**IDU **Yes vs. No	**6.64**	**9.76**	**4.86-9.07**	**5.76-16.54**
**MSM **No vs. Yes	**2.99**		**2.09-4.27**	

*Separate model for males and females. Models include age at HIV, IDU, aboriginal ethnicity, Health Authority and MSM (for males only)

Diagnosis of HCV before HIV in univariate analysis of co-infected individuals was significantly associated with older age, female sex, reported IDU and not being identified as MSM in males (Table 3). In the separate multivariate logistic regression models for males and females, HCV diagnosis before HIV was significantly associated with age and IDU in both sexes (Table 4). The majority of persons co-infected reported IDU as the major risk factor; a small proportion (6.2%) of co-infected males reported both MSM and IDU and 5.4% reported MSM only (Table 5).

**Table 3:** Order of virus identification for individuals with HIV/HCV co-infection, January 1^st ^1995- December 31^st^, 2008

	HIV first (%)		HCV first (%)		Unadjusted OR		95% Confidence Limits	
**N**	379		876					
								
**Age at HIV**					**1.05**		**1.04-1.07**	
Mean	34.3		38.6					
Std	9.05		9.31					
Range	15-61		18-75					
								
**Sex**								
Male	265 (69.9)		559 (63.8)		1.00			
Female	112 (29.6)		313 (35.7)		**1.33**		**1.02-1.72**	
Transgender	1 (0.3)		3 (0.3)					
Unknown/ missing	1 (0.3)		1 (0.1)					
								
**Ethnicity**								
Non-Aboriginal	234 (61.7)		570 (65.1)		1.00			
Aboriginal	79 (20.8)		244 (27.8)		1.27		0.94-1.70	
Unknown/ missing	66(17.4)		62 (7.1)					
								
**Health Authority**								
Vancouver Coastal	191 (50.4)		335 (38.2)		1.00			
Fraser	108 (28.5)		205 (23.4)		1.08		0.81-1.45	
Interior	21 (5.5)		65 (7.4)		**1.77**		**1.05-2.98**	
Northern	10 (2.6)		74 (8.5)		**4.22**		**2.13-8.36**	
Vancouver Island	45 (11.9)		195 (22.3)		**2.47**		**1.71-3.58**	
Missing	4 (1.1)		2 (0.2)					

	**Male**	**Female**	**Male**	**Female**	**Male**	**Female**	**Male**	**Female**
**MSM**								
Yes	47 (17.7)	-	55 (9.8)	-	1.00			
No	218 (82.3)	-	504 (90.2)	-	**1.98**		**1.30-3.01**	-
								
**IDU**								
Yes	169 (63.8)	81 (72.3)	474 (84.8)	266 (85.0)	**3.17**	**2.17**	**2.26-4.46**	**1.28-3.62**
No	96 (36.2)	31 (27.7)	85 (15.2)	47 (15.0)	1.00	1.00		

**Table 4:** Adjusted ORs (multivariate logistic regression) of HCV first vs. HIV first

	Adjusted OR*		95% Confidence Limits	
	**Male** **(N = 726)**	**Female** **(N = 392)**	**Male**	**Female**

**Age group at HIV**				
25-34 years vs. 15-24 years	1.79	**2.20**	0.85-3.77	**1.15-4.19**
35-44 years vs. 15-24 years	**3.22**	**3.73**	**1.56-6.66**	**1.87-7.42**
45+ years vs. 15-24 years	**7.11**	**8.15**	**3.24-15.59**	**2.88-23.09**
**Health Authority (HA) vs. Vancouver Coastal HA**				
Fraser HA	1.16	1.02	0.76--1.77	0.54-1.90
Interior HA	**2.12**	1.61	**1.00-4.49**	0.57-4.54
Northern HA	2.57	**5.83**	0.99-6.72	**1.64-20.78**
Vancouver Island HA	**2.67**	1.18	**1.57-4.54**	0.61-2.28
**Aboriginal **Yes vs. No	1.35	1.27	0.85-2.15	0.76-2.12
**IDU **Yes vs. No	**2.83**	**2.25**	**1.84-4.37**	**1.15-4.39**
**MSM **No vs. Yes	1.48		0.90-2.44	

*Separate model for males and females. Models include age at HIV, IDU, aboriginal ethnicity, Health Authority and MSM (for males only)

**Table 5:** Major risk factor categories HIV/HCV co-infection, BC January 1, 1995- December 31^st^, 2008

MALES				
	HCV first (%) n = 559	HIV/HCV Same (%) n = 325	HIV first (%) n = 265	All n = 1,149
IDU	436 (78.0)	247 (76.0)	154 (58.1)	837 (72.8)
MSM	17 (3.0)	13 (4.0)	32 (12.1)	62 (5.4)
MSM/IDU	38 (6.8)	18 (5.5)	15 (5.7)	71 (6.2)
Heterosexual Contact	35 (6.3)	26 (8.0)	33 (12.1)	94 (8.2)
Nil identified/Unknown	28 (5.0)	18 (5.5)	28 (10.6)	74 (6.4)
Other	5 (0.9)	3 (0.9)	3 (1.1)	11 (1.0)
				
**FEMALES**				

	**HCV first (%)** **n = 313**	**HIV/HCV Same (%)** **n = 120**	**HIV first (%)** **n = 112**	**All** **n = 545**

IDU	266 (85.0)	100 (83.3)	81 (72.3)	447 (82.0)
Heterosexual Contact	32 (10.2)	14 (11.7)	22 (19.6)	68 (12.5)
Nil identified/Unknown	13 (4.2)	5 (4.2)	9 (8.0)	27 (5.0)
Other	2 (0.6)	1 (0.8)	0 (0.0)	3 (0.6)

## Discussion

This study provides important new information from a population-based sample of HIV and HCV tested individuals. We are able to provide a robust estimate of those who have been infected with both HCV and HIV, the order of virus identification and associated risk factors. Previous studies which assess co-infection have typically been cross-sectional surveys or longitudinal cohorts following persons who inject drugs or are MSM and therefore results are more likely to be population specific or affected by selection bias.

This study uses anti-HCV positivity to define HCV infection and co-infection. We recognize that approximately 25% (range 15% to 45%) of infected individuals will clear infection spontaneously [23]. As HCV-RNA testing was available only in a subgroup of anti-HCV positive individuals, we are unable to confirm active HCV infection in individuals diagnosed with HCV. Therefore our study overestimates the number of individuals who are actually HCV/HIV co-infected.

The HIV/HCV co-infection rate in our HIV seropositive sample is high compared to other studies [1]; over half of persons with HIV who were tested for HCV were found to be HCV positive. In a population sample persons who are at highest risk for HCV infection such as IDU may be more likely to be tested and therefore over-estimate co-infection. IDU was independently associated with HCV co-infection in both sexes, but reported aboriginal ethnicity was significant in females only. Among a cohort of IDU aged <30 years in Vancouver, coinfection at baseline was independently associated with being female, aboriginal ethnicity, involvement in sex trade, older age, greater number of years injecting [4]. Our study population is based on persons who are seropositive for HIV rather than IDU, therefore the results are not directly comparable.

Our study sample does not represent systematic sampling of the population, but is based on all laboratory confirmed HIV infected cases during the study period that were then linked to individuals who had undergone HCV serologic testing. The sample is therefore biased to those who are offered and undergo testing. Although there is an effort to make testing acceptable and available in clinics and through outreach throughout BC, there may be geographic differences.

To reduce the bias for order of detection, 1995 was chosen as the start date, excluding only 11 HIV cases. HCV has been reportable and testing routinely available since 1992; thus 32,428 individuals prior to 1992 were excluded. However, these would be included if HCV testing occurred at a later date. In addition, 80 positive HIV cases were excluded as aged <15 years or non-BC residence.

Between January 1995 to April 1997 anti-HCV testing involved screening with the slightly less sensitive second generation anti-HCV test followed by immunoblot confirmation. Since April 1997 to the end of the study period all testing involved screening with the more sensitive 3^rd ^generation anti-HCV assay and confirmation by a second manufacturer's enzyme immunoassay. Only specimens reactive by both manufacturers' tests were considered to be anti-HCV reactive. Therefore it is unlikely that changes in anti-HCV serological testing over the course of the study impacted HCV diagnostic accuracy.

Our results may have been affected by changes in data quality over time. HIV became a reportable infection in BC in 2003; at this time enhanced regional public health follow-up of individuals having a new positive HIV test was established resulting in improved ascertainment of ethnicity and exposure categories. The quality of identifier data used for data linkage has also changed over time, and is most complete after 2003. Therefore co-infection of HIV cases identified prior to 2003 may be under-reported, or bias introduced in subgroup analyses.

The use of partial identifiers, pseudonyms, or non-nominal testing (a testing option introduced in 2003 for HIV) also affects our ability to link data accurately. In studies which require data linkage, some vulnerable and high risk populations are more likely to be excluded due to lack of personal identifiers or difficulty in obtaining risk factor information. Therefore our study may underestimate the number of HIV cases who are HCV co-infected.

The HIV positive population that had not been tested for HCV differed by age, sex and ethnicity; therefore, our study results cannot be generalized beyond the linked population. As IDU is a well known transmission route for HCV, persons who use intravenous drugs may be more likely to be offered and accept HCV testing. Thus we may have overestimated the odds of IDU in co-infection, but the strength of the association is clear.

Our study population uses an HIV dataset to drive the linkage therefore the major exposure categories were determined according to HIV risk rather than HCV risk. Although the risk factors are similar, they may change over time and differ at the time of HCV co-infection diagnosis from those identified at the time of HIV diagnosis. Ascertainment of accurate exposure categories such as IDU and MSM may not be elicited during follow-up due to social desirability bias.

Our study reflects the initial diagnosis of HIV and HCV; however it cannot determine when the infection occurred nor confirm the order of infection. Over 80% of persons acutely infected with HCV are asymptomatic and may have undiagnosed HCV infection for decades [24]. The order of virus detection will also be affected by availability and patterns of testing as well as the patient's willingness to be tested for HCV and/or HIV.

If testing for both viruses occured within 2 weeks we considered this to be simultaneous identification. Patients may have been tested for HIV and HCV due to reported risk factors, or the testing of second virus may immediately follow identification of the first. Some patients maybe diagnosed with co-infection as part of a diagnostic work up for mild or moderate disease, or when they present in the emergency room with opportunistic infections [25].

Although the predominant risk factor for HCV infection reported in HIV seropositive men is IDU [26,27], acquisition of HCV in HIV seropositive MSM without a history of IDU is increasingly reported [28]. In this group, HCV seroconversion has been associated with other sexually transmitted infections such as syphilis or lymphogranuloma venereum and/or sexual practices causing mucosal damage such as fisting [28-30]. We found 5.4% of co-infected males reported MSM and no history of IDU. This is likely an underestimate due to lack of HCV testing in this group but suggests that sexual transmission of HCV is occurring in this population. MSM who are HIV infected should be informed that they may be at risk for HCV even if they do not use intravenous drugs and offered testing, especially if considering HIV treatment [13-15].

HCV was identified first in about half of those who were co-infected, with a median time to HIV detection of about 3 1/2 years. This highlights the need for effective public health follow-up when HCV is identified. HCV is more easily transmitted percutaneously and has a higher prevalence in IDU than HIV. Miller et al studied co-infection in a cohort of young IDU and found accessing methadone maintenance therapy in the previous 6 months was protective from seroconversion of the second infection [4]. Identification of HCV provides an opportunity for public health interventions including testing for other blood borne pathogens such as HIV, and offering appropriate immunizations and harm reduction measures. Engagement through harm reduction such as providing clean needles and other drug use paraphernalia and methadone maintenance can decrease the risk of subsequent HIV infection [31].

## Conclusion

The high prevalence of co-infection supports the need for HCV screening in HIV positive populations. It is important to ensure HIV and HCV testing is accessible and offered in gender and culturally appropriate manner. The ability to link BC public health and laboratory HIV and HCV information provided a unique opportunity to explore demographic and risk factors associated with HIV/HCV co-infection in a large population-based sample. Over half of persons with HIV infection who were tested for HCV were shown to be anti-HCV positive, and half of these had HCV identified first with HIV identification a median 3.5 years later. This study highlights the importance of public health follow-up and harm reduction measures for people identified with HCV as a means to prevent subsequent HIV infection.

## Competing interests

The authors declare that they have no competing interests.

## Authors' contributions

JAB participated in the design and coordination of the study and drafted the manusript. AY and PHK extracted, manipulated, and prepared the data. JJS provided statistical advice and reviewed the analysis of the data. MaK, MA, and MG contributed with the interpretation of the data. MeK took part in the planning of the study and oversaw the data. All authors read and approved the final manuscript.

## Pre-publication history

The pre-publication history for this paper can be accessed here:

http://www.biomedcentral.com/1471-2458/10/225/prepub
